# Psychotic Features Abated with Vitamin D Treatment in a Patient with Major Depressive Disorder

**DOI:** 10.1155/2020/2046403

**Published:** 2020-06-19

**Authors:** David C. Fipps, Elisabet Rainey

**Affiliations:** University of South Carolina, Greenville Prima Health, Upstate 701 Grove Road Greenville, SC 29605, USA

## Abstract

**Introduction:**

Vitamin D deficiency is common in psychiatric populations, and vitamin D has been used as an add-on medication in major depressive disorder. *Case Report*. Here, we present the case of a 49-year-old man diagnosed with major depressive disorder, severe, with psychotic features, who was treated with antidepressants and underwent multiple antipsychotic trials. The patient was found to have low serum levels of vitamin D. During treatment with vitamin D, serum levels normalized and the patient's psychotic symptoms abated.

**Conclusion:**

Ensuring adequate vitamin D levels could be a method of focus for the augmentation or treatment of psychotic features in major depressive disorder.

## 1. Introduction

The twelve-month prevalence of major depressive disorder (MDD) in the United States has been estimated to be approximately 7% [1]. Forty percent of individuals typically enter recovery phase within three months of onset, and 80% typically have entered recovery by one year [2]. However, lower recovery rates are seen during certain circumstances, including prominent anxiety, personality disorders, high symptom severity, and psychotic features [3]. The diagnostic and statistical manual of mental disorders, fifth edition (DSM-5), indicates that the specifier “with psychotic features” is appropriate when there are delusions and/or hallucinations that are present at any time within the depressive episode and discusses differentiating between the mood congruencies of these symptoms [4]. Recent literature indicates that psychotic depression holds a lifetime prevalence between 0.35% and 1% with higher rates in older age, with no differences in gender distribution [5]. Treatment for major depressive disorder, severe, with psychotic features (MDDPF) typically includes antipsychotic medications in addition to antidepressants [3]. However, due to the severity of MDDPF, it also qualifies for procedural interventions, such as electroconvulsive therapy (ECT), as an additional first line treatment option [3].

The fulminant etiology of psychosis is not well understood and likely includes multiple underlying causes with many contributing and exacerbating factors. There are many hypotheses surrounding the pathophysiology of psychosis, with a prominent and traditional understanding revolving around that of a dopaminergic origin. Auditory hallucinations are most prevalent in psychotic disorders such as schizophrenia and schizoaffective disorder, but they can clearly occur in other psychiatric disorders, such as MDD, bipolar disorder, and substance use disorders and even in the general population [6]. Epidemiological studies have estimated the prevalence of auditory hallucinations to be between 5% and 28% in the general population [7–10]. One study found that 25% of individuals reporting hallucinatory experiences met the diagnostic criteria for a psychotic disorder; however, that leaves 75% of people experiencing auditory hallucinations from a completely different etiology [11].

## 2. Case Report

### 2.1. Patient Information

Mr. V was a 49-year-old married, Caucasian, college-educated, male, disabled veteran with major depressive disorder, severe, with mood incongruent psychotic features. Note that the information that could be deemed as identifiable characteristics has been changed to ensure patient privacy. None of these changes distort the scientific findings or clinical relevance of this case report.

### 2.2. Case Timeline

**Figure 1:**
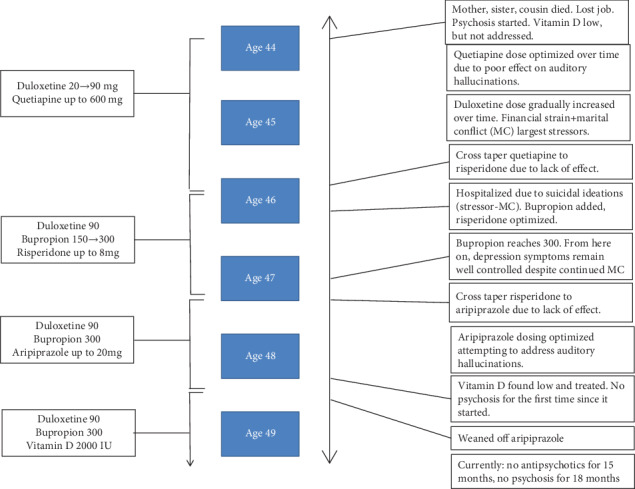
Case timeline.

Mr. V had struggled with depression for roughly 20 years. During the first 15 years of depression, the patient's symptoms had shown improvements with both psychopharmacologic and psychotherapeutic interventions; however, for the past five years the symptoms had been more difficult to manage as he began to experience psychotic symptoms. The following case presentation is a summary of Mr. V's treatment over these five years. It is split into two sections: (1) Care Prior to This Author and (2) Care with This Author. A timeline figure has been included for clarity (see Figure 1).

### 2.3. Care prior to This Author

Mr. V was referred to the psychiatry clinic from his primary care physician in the context of worsening depression symptoms and auditory hallucinations. The initial laboratory workup from the primary care physician included complete blood count, comprehensive metabolic panel, thyroid panel, lipid panel, folate, vitamin B12, and vitamin D. All results were within normal limits with the exception of mixed hypercholesterolemia and low vitamin D concentrations (23.8 ng/mL; reference level ≥ 28.9 ng/mL). The patient also attained a computed tomography scan of his head, which showed no abnormalities. No electroencephalogram was attained in the initial workup. Physical exam findings were unremarkable.

In the initial appointment with his first psychiatrist in this timeframe, the patient was evaluated using the Structured Clinical Interview for DSM-5 [12]. According to this evaluation, the patient demonstrated significant depressive symptoms with anhedonia, but the most prominent symptoms included lack of motivation, very low energy level, fatigue, and psychomotor retardation. The patient was screened for bipolar disorder, anxiety disorders, obsessive compulsive disorder, posttraumatic stress disorder, and personality disorders, all of which were negative. He did not see combat in the military, and his disability compensation was secondary to a lower back injury from his previous civilian job. The patient was able to clearly recollect his first experience of auditory hallucinations at the age of 44 years after the deaths of multiple family members and losing his job. The patient's depression was severe at that time, and he began isolating himself from the outside world. He described hearing indistinct muffled voices, as if a group of people were talking in a different room. He stated that these voices were very difficult to distinguish and low enough that he could not understand their words. This occurred predominantly at night, though it did not have any hypnagogic or hypnopompic correlation. The patient denied and did not demonstrate any behavior characteristic of experiencing delusions, disorganized speech, or disorganized or catatonic behavior. The diagnosis of MDDPF was made, and the patient was started on duloxetine 20 mg/day and quetiapine 50 mg/day. It appears that the patient was lost to follow-up with primary care, and the psychiatrist did not address his vitamin D level; therefore, this value was left untreated for years.

Though Mr. V did not maintain his primary care appointments, he was mostly adherent to his psychiatric follow-up. Over the next few years, he underwent many psychopharmacologic changes in attempts to address his depression and his psychotic features (see Figure 1).

According to the documentation, the follow-up evaluations included structured interviews for symptomatic assessments and patient health questionnaire-9 (PHQ-9) [13] depression scores. Mr. V's depression symptoms fluctuated throughout the years often linked to stressors, such as financial difficulties, marital conflict, and deaths in the family. However, with each antidepressant dosing optimization, these symptoms appeared to steadily improve without any direct correlation of change in his psychotic symptoms. In fact, it appeared there was barely any correlation with his psychotic symptom severity and the severity of his depression symptoms. For example, there was a time of clear depression symptomatic exacerbation (to the point of suicidal ideations requiring inpatient hospitalization); however, this did not change the intensity or severity of his hallucinatory reports. On the other hand, there was a separate encounter where he voiced an excellent response to his depression treatment, but we noted that his voices were more prominent, i.e., not louder or more frequent, but more clear and understandable (described as brief, infrequent, and attention seeking exclamations). Interestingly, documentation indicates that when the patient would present saying both his depression and voices were improving (but not gone), he would quantify this by saying: “I can tell when I'm better, because I'm able to go outside more and work in the yard, enjoy the sunlight”.

The patient's duloxetine dose was gradually increased over time to 90 mg/day. During the aforementioned hospitalization, the patient was augmented with bupropion, which was eventually optimized to 300 mg/day over time. Upon reaching these doses, the patient sustained a level of stability with his depression symptoms despite continued stressors and despite continued auditory hallucinations.

The patient's auditory hallucinations over these years ranged from the originally described muffled voices to more complex and clear hallucinations. At times, he heard doorbells when no one was at his door and faint sounds similar to footsteps in his hallway when he could clearly see no one in the hallway. The strongest and most disturbing hallucinations included multiple instances of a clear single, nonrecognizable, male voice using one-word exclamations like, “hey,” “look,” “what?” and the patient's name, typically occurring when the patient was alone or not actively engaged in a conversation. These hallucinations, and their continuation despite dosing optimization, prompted multiple antipsychotic trials.

Mr. V's aforementioned quetiapine dose was gradually optimized to 600 mg/day before cross titrating to risperidone due to lack of effect. The risperidone dose was pushed up to 8 mg/day with little to no effect on his auditory hallucinations. He was then cross titrated to aripiprazole, and it was eventually dosed up to 20 mg/day. Each antipsychotic trial lasted between 9 and 18 months before switching due to little or no change in his auditory hallucinations (see Figure 1).

Prior to being inherited by this author, two separate psychiatrists evaluated the patient and agreed that the most likely diagnosis was MDDPF. They both indicated in their documentation that there was a potential that this could be schizoaffective disorder considering the presence of psychotic features in the absence of depressive symptoms; however, neither was confident with a primary psychotic disorder diagnosis considering the patient's age, unusual timing, description, and level of functioning. ECT was considered, but the patient declined this intervention.

### 2.4. Care with This Author

Upon inheriting this patient, who then was prescribed duloxetine 90 mg/day, bupropion 300 mg/day, and aripiprazole 20 mg/day, he was able to voice a fairly cogent summary of the above historical symptom progression and responses to medications. In our first encounter, the patient's PHQ-9 was 4 out of 27 (2 for trouble falling asleep and 2 for feeling tired and having little energy) and Generalized Anxiety Disorder-7 (GAD-7) [14] score was 2 out of 21 (1 for not being able to control worry and 1 for trouble relaxing, which he directly correlated to timeframes of having auditory hallucinations). By this time, the patient noted that he heard the muffled voices and footsteps almost nightly and he would hear the short exclamation auditory hallucinations 3-4 times a week and 2-3 times a night. He voiced a clear satisfaction with his depression treatment, and by this time, he viewed his auditory hallucinations as an annoyance rather than a frightening experience. He knew these experiences were hallucinations rather than people being in his house, but he still wished to continue to pursue treatment for these symptoms. The patient had been on aripiprazole for a total of nine months and had been at 20 mg/day for three months. Discussions ensued for the next step of antipsychotic treatment, such as a first-generation antipsychotic. He was very near the due date for antipsychotic laboratory screening testing; thus, it was also decided to include extra screening panels to allow a more comprehensive evaluation of potential rarities that could cause psychosis (human immunodeficiency virus infection, systemic lupus erythematosus, syphilis, folic acid, vitamin D, etc.). All the laboratory results were unremarkable with the exception of hypercholesterolemia and low 25-hydroxyvitamin D level, now at 22.3 ng/mL (reference level ≥ 28.9 ng/mL).

The patient was not interested in changing his antidepressant medications due to his current symptomatic stability, but was willing to start vitamin D replenishment. He also preferred to make one change at a time, no matter how small; thus, he requested waiting on changing his antipsychotic until after his vitamin D was started. He was started on 2000 IU/day of cholecalciferol with behavioral activation discussions, particularly referring to getting out into the sunlight more and/or obtaining a light box.

After one month of follow-up, the patient had not been able to get out of the house more than usual and he had not bought a light box. However, he voiced for the first time in 3-4 years that he has had complete resolution of the psychotic features. The patient strongly felt this to be a direct effect of vitamin D treatment, which was further strengthened when recheck of vitamin D was within normal limits. The treatment team originally considered this symptomatic improvement to be coincidental, and discussions continued for future plans of change to another antipsychotic, particularly because the patient began voicing concerns for akathisia from aripiprazole. On further follow-up appointments, the patient continued to demonstrate sustained remission of his psychotic features despite the continually decreasing aripiprazole dose to address his akathisia. This sustained remission continued, and upon reaching subtherapeutic dosing of aripiprazole, the decision was made to attempt a trial of complete discontinuation of all antipsychotic treatment. At the time of this writing, the patient has been off all antipsychotics for 15 months and has had complete absence of psychotic features for 1.5 years with duloxetine, bupropion, and cholecalciferol at doses noted above. The patient's vitamin D levels have remained within normal limits during this timeframe.

## 3. Discussion

This case brings forth multiple aspects regarding vitamin D and its role for psychiatric symptoms and treatment. First, we will discuss the basics of vitamin D and its prevalence in the psychiatric community. Second, we will correlate vitamin D treatment with different types of psychiatric disease and dopaminergic functioning. Lastly, we will comment on how this case also brings forth the importance of assessment of somatic health in psychiatry.

Vitamin D is naturally found in eggs and fish and is often artificially added to dairy products [15]. The body will produce vitamin D from cholesterol in the presence of ultraviolet B light from the sun [16]. Low vitamin D levels are associated with an increase in all-cause mortality in the general population independent of race or gender [17]. Low vitamin D is quite common, particularly in the psychiatric community. A study focusing on a chart review of 544 adult psychiatric patients in Chicago, Illinois, found that 75% had vitamin D insufficiency (defined as <30 ng/mL) with a mean level of 22 ng/mL [18]. Risk factors for low vitamin D include low sunlight exposure (indoor work, dark skin tone, northern geographic local, winter months, and bedridden status), chronic illness, obesity, and advanced age [19]. In our patient, it is important to point out that the times of his documented improvement in both his depression and psychotic symptoms (before prescribing vitamin D) occurred during times when he was able to get out into the sunlight more often. Common supplementation forms include vitamin D2 (ergocalciferol) and D3 (cholecalciferol), and current evidence indicates that D3 supplementation may be more efficient for raising 25-hydroxyvitamin D levels than D2 [15]. Repletion can be accomplished in patients with 25-hydroxyvitamin D levels lower than 50 ng/mL with 600-4000 IU/day of cholecalciferol (D3) [16]. Vitamin D toxicity is very rare and typically occurs after prolonged periods of very large doses (>10,000 IU/day) [10].

From a clinical standpoint, most literature regarding vitamin D usage in psychiatric illness is focused on MDD. Epidemiological data shows that vitamin D deficiency is associated with an 8% to 14% increase in depression [20]; however, the literature findings regarding vitamin D supplementation in depression have been conflicting at best. For example, two systematic reviews found that studies on vitamin D for MDD were inconclusive [21, 22] while another meta-analysis from the same year found a statistically significant effect of vitamin D supplementation on depression scores, comparable to the magnitude of the effect of typical antidepressant medications [23]. In regard to psychosis, there have been studies showing correlations with vitamin D and psychotic symptoms. In one meta-analysis including 2,804 participants, those who were vitamin D deficient were 2.16 times more likely to have schizophrenia, and over 65% of the participants with schizophrenia were vitamin D deficient [24]. A cross-sectional analysis of mentally ill adolescents (age 12-18 years) requiring inpatient or partial hospitalization found that those with vitamin D deficiency were 3.5 times more likely to have hallucinations, paranoia, or delusions [25].

There also appears to be some interplay between that of vitamin D and dopaminergic functioning. Animal studies have shown that adult female rats exposed to vitamin D deficiency have an increase in dopamine transporter density in the caudate putamen and increased dopamine binding affinity in the nucleus accumbens [26]. There have also been a number of studies showing that vitamin D-deficient rats are selectively sensitive to postsynaptic dopamine blockade to haloperidol [27]. Lastly, in the absence of vitamin D during development, there are early reductions in the expression of crucial specification factors for dopaminergic neurons [28] and reductions in enzymes involved in dopamine turnover with accompanying alterations in dopamine metabolites [29]. Emerging data has linked changes in the dopaminergic system to the pathophysiology of depression. Many of the symptoms seen in depression such as anhedonia and amotivation have been more consistently associated with dysfunctions in dopamine versus the traditional serotonergic theory [30–32]. These particular dopamine-related symptoms were quite prominent in our patient's depression.

However, the dopaminergic theory of influence on psychiatric symptoms has been most traditionally linked to that of psychosis, particularly the long taught conceptualization of the dopaminergic mesolimbic and mesocortical pathways for positive and negative psychotic symptoms, respectively. Considering the previously described role of vitamin D in dopaminergic functioning, it would be logical to consider vitamin D deficiency influencing this patient's psychotic symptoms. This is particularly of note considering that the patient's auditory hallucinations did not always coincide with the severity of his depressive symptoms, and the symptoms improved (and sustained) with normalization of his vitamin D level. With that said, the etiology of depression and psychosis is likely multifactorial with vitamin D deficiency being only one piece of many potential pathophysiologic underpinnings and contributions.

In closing, this case also brings forth the importance of assessment of somatic health in psychiatric clinics. Considering our field is one that highly values the therapeutic alliance and a bond of trust between physician and patient, it is not uncommon for physical complaints to be voiced and/or discovered by the psychiatric physician. This does not mean psychiatrists should take on a role outside of the scope of their practice; however, there is a great interplay between comorbid somatic conditions and that of our psychiatric presentations. In fact, many general medical conditions hold a bidirectional comorbidity with many psychiatric illnesses. For example, MDD has been shown to increase the risk of coronary artery disease [33], cancer [34], Alzheimer's disease [35], diabetes mellitus [36], etc. Ensuring appropriate assessment and focus on somatic health in psychiatric clinics can be of great benefit to our patients, as we saw in this case where the symptomatology and need for treatment changed over time considering the changes in vitamin D level.

## 4. Conclusion

This case report validates that low vitamin D could be a cause and/or an exacerbating factor that could contribute to psychotic symptoms. Therefore, vitamin D replenishment could be an appropriate augmentation strategy in MDDPF, particularly if the vitamin D level is found to be low.
